# Case report: Bone marrow metastasis and bone marrow necrosis occurring 11 years after ductal carcinoma *in situ* of the breast

**DOI:** 10.3389/fonc.2024.1473896

**Published:** 2024-12-23

**Authors:** Shuting Zhang, Zhonghai Du, Jun Wu, Xiaoli Zhang, Wei Dong

**Affiliations:** ^1^ Graduate Institute, Shandong University of Traditional Chinese Medicine, Jinan, Shandong, China; ^2^ Department of Oncology, Weifang Hospital of Traditional Chinese Medicine, Weifang, Shandong, China; ^3^ Department of Pathology, Weifang Hospital of Traditional Chinese Medicine, Weifang, Shandong, China

**Keywords:** ductal carcinoma in situ, bone marrow metastasis, bone marrow necrosis, bone marrow biopsy, ^18^F-FDG PET/CT, BRCA1, triple-negative breast cancer

## Abstract

Ductal carcinoma *in situ* (DCIS), a noninvasive breast cancer, rarely metastasises to distant locations. When the initial lesion is stable, bone marrow metastasis (BMM) and bone marrow necrosis (BMN) are even less common. Here, we report the case of a 47-year-old female patient who underwent localized surgery and radiotherapy for right-sided DCIS. The patient also had a mutation in the breast cancer susceptibility gene 1 (*BRCA1*, OMIM: 113705) and tested positive for the progesterone and estrogen receptors. After 11 years of disease-free survival, the patient developed severe thrombocytopenia, anemia, fever, malaise, generalized multifocal pain, and irregular vaginal bleeding. A nodule was later found in the right axilla, and a postoperative biopsy revealed tumor cells from the breast. After three bone marrow biopsies, Positron Emission Tomography, ^18^F-fluorodeoxyglucose, positron emission tomography, computed tomography (^18^F-FDG PET/CT) scans, and other examinations, she was finally diagnosed with breast cancer BMM and BMN (stable primary lesion without bone metastasis). Despite symptomatic supportive treatment, the patient ultimately died rapidly as her condition deteriorated. In this case, we explored the possible mechanisms of BMM in this patient with DCIS by reviewing the literature related to this case and discussing the heterogeneous clinical presentation and pathologic phenotype. The diagnostic and therapeutic course of this case was extremely challenging. This suggests to clinicians that regular checkups and monitoring are necessary, even if the rate of distant metastasis from DCIS is low.

## 1 Introduction

Breast ductal carcinoma *in situ* (DCIS) is characterized by abnormal epithelial cells restricted within the mammary ducts, surrounded by intact myoepithelial cells and basement membrane (1). It accounts for about 25% of new breast cancer diagnoses and is considered non-invasive as long as it remains within the ducts (2–4). However, low-grade DCIS has the potential to progress into invasive cancer, with rare occurrences of distant metastasis reported at only 0.14% (5, 6). The most common sites of distant metastasis in breast cancer are the lungs, bones, liver, and brain (7–9). Symptomatic bone marrow metastasis (BMM) is an exceptionally rare complication in DCIS, with most cases arising in the context of invasive breast cancer (10). In addition, asymptomatic bone marrow metastases are reported in 20-30% of patients with early-stage breast cancer and are usually caused by disseminated tumor cells (DTCs) that are clinically insignificant (10, 11). Under specific conditions (e.g., changes in the tumor microenvironment caused by systemic inflammation), these dormant cells may be reactivated. When circulating tumor cells (CTCs) invade the bone marrow, replacing normal tissue and causing symptoms like anemia, thrombocytopenia, and coagulation abnormalities, it’s called symptomatic bone marrow involvement (10, 12). Bone marrow necrosis (BMN) is a rare clinicopathologic condition, often overlooked in living patients, characterized by extensive necrosis of hematopoietic tissues and stroma, with symptoms including bone pain, fever, and hematologic abnormalities such as anemia and thrombocytopenia (13, 14). Common symptoms of bone pain are due to inflammation and increased pressure within the bone caused by the metastasis or necrosis of bone marrow, which activates the peripheral sensory nerve endings within the bone marrow by releasing inflammatory mediators and mechanical compression or deformation (15). Malignant tumors are the main cause of BMN, accounting for approximately 90% of BMN cases, with malignant diseases of the hematopoietic system accounting for 60%, and extensive BMN secondary to solid tumors are rare and are usually an end-stage manifestation of symptomatic BMM (14). This report describes a rare case of a female patient who developed symptomatic BMM and eventually BMN 11 years after breast-conserving surgery and radiotherapy. By reviewing the relevant literature, we attempted to analyze the pathological mechanism, clinical characteristics, and treatment options to provide a reference for diagnosing and managing similar cases.

## 2 Case description

**Figure 1:**
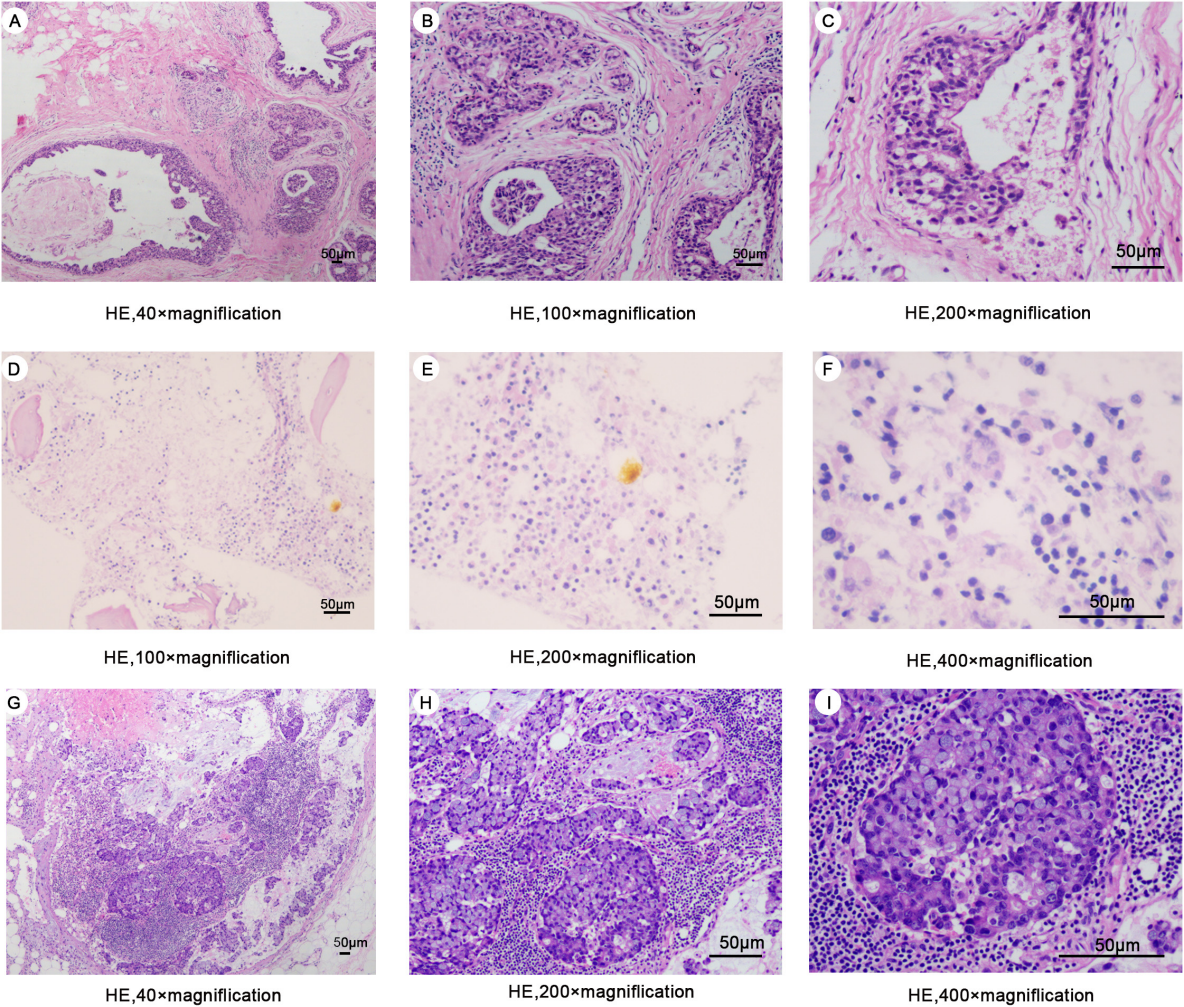
**(A–C)** Hematoxylin-eosin staining of surgical excision specimens of the right breast in 2013 **(D–F)** Hematoxylin-eosin staining of bone marrow biopsy specimens in 2024. **(G–I)** Hematoxylin-eosin staining of a specimen of the right axillary mass in 2024.

**Figure 2:**
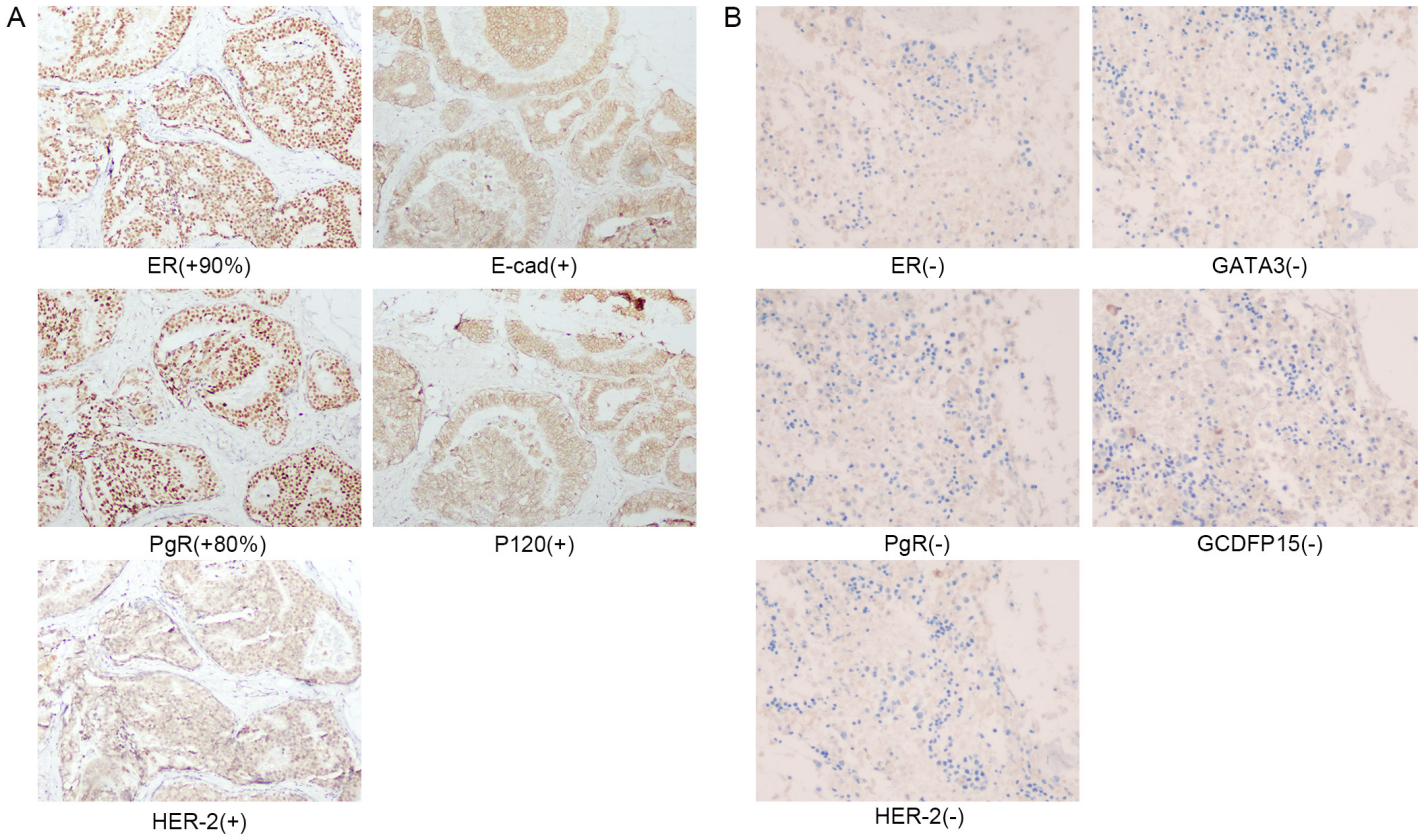
**(A)** Immunohistochemical staining of right breast surgical excision specimen in 2013. **(B)** Immunohistochemical staining of the second bone marrow biopsy in March 2024.

A 47-year-old woman presented to our hospital on February 24, 2024, with fatigue and neck, shoulder, and back pain for the past month. She had been admitted to the hospital in January 2013 for “bloody right nipple discharge for two months.” At that time, she underwent a segmental excision of the right breast lesion and a biopsy of the anterior lymph nodes. The lesion was completely excised with negative margins. Pathologic findings showed non-invasive right breast ductal carcinoma *in situ* (**Figures 1A–C**). Immunohistochemistry detected carcinoma *in situ* estrogen receptor (ER) (+90%), progesterone receptor (PgR) (+80%), human epidermal growth factor receptor 2 (HER2) (+), E-cad (+), 34βE12 (+), P120 (membrane +), Ki-67 (<10%), P53 (+<10%), and SMA (+) (**Figure 2A**). The breast cancer was graded as pTisN0M0. Genetic testing showed a positive result for the S1 locus of the *BRCA1* gene. The patient refused further right mastectomy. Eight weeks after surgery, the patient underwent conformal radiotherapy to the right breast at a dose of DT: 4800cGy/25f, with enhanced DT to the right breast surgical area: 800cGy/4f. The patient was discharged with a good recovery. Despite being advised to take tamoxifen (20 mg daily) due to her hormone receptor-positive (HR+) breast cancer, the patient stopped the medication a few days after discharge and did not attend regular follow-up examinations.

**Figure 3:**
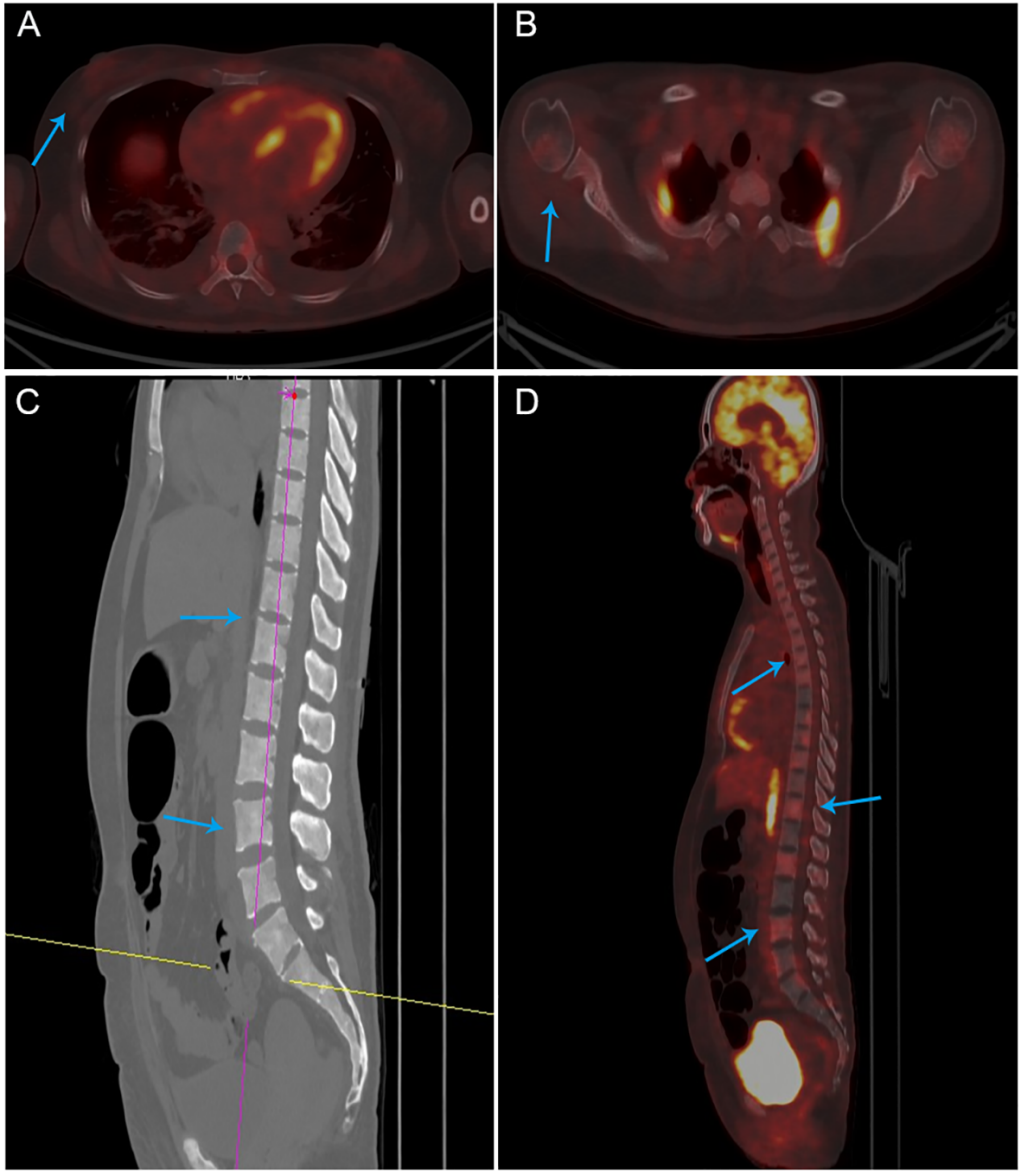
PET-CT images **(A, B)** There was no abnormal metabolism in the area of the right breast surgery or the axilla. **(C, D)** Diffuse hyperdensity in the medullary cavity of the bone, no destruction of the bone cortex.

On this admission, the patient reported heavy and prolonged menstrual flow for the last two months. There was a history of Coronavirus disease 2019 (COVID-19) infection in previous months. The patient denied any family history of genetic disorders or malignant tumors. The breast examination was unremarkable, showing no changes in the right breast or the scar area from the right axillary surgery. Routine blood tests showed platelets (16 × 10^9^/L) and hemoglobin 51 g/L. Tumor markers showed CA153 at 45 U/L, while CEA and CA125 were within normal ranges. On March 2, the patient developed a fever accompanied by vaginal bleeding of about 40 ml, and platelets continued to fall (12 × 10^9^/L). Novel Severe Acute Respiratory Syndrome Coronavirus 2 (SARS-CoV-2) Nucleic Acid Test Returns Positive. Two days later, her plasma D-dimer increased to 55.29 mg/L. By March 7, her alkaline phosphatase had abnormally increased to 2398 U/L. Computed tomography (CT) scans of the craniocerebral, thoracic, abdominal, and pelvic regions, along with breast MRI, did not reveal any recurrence or other metastatic foci of breast cancer. Subsequent ^18^F-FDG PET/CT examination showed no abnormal metabolism in the area of the right breast surgery or elsewhere in the body (**Figures 3A, B**), diffuse hyperdensity in the bone marrow cavity, mainly located in the medial skeleton, suggestive of BMM, and no destruction of the bone cortex. (**Figures 3C, D**). Serum immunofixation electrophoresis revealed normal serum immunoglobulin G, A, and M levels, reducing the likelihood of multiple myeloma.

Upon the patient’s admission to the hospital, we performed the first bone marrow aspiration biopsy. The biopsy results indicated metastatic cancer in the marrow. Immunohistochemistry results showed ER (-), PgR (-), and HER2 (-). We conducted another bone marrow aspiration biopsy two weeks later to clarify the diagnosis. We examined two pieces of bone marrow tissue obtained from different puncture sites in the patient’s iliac bone, and both results confirmed BMN (**Figures 1D–F**). The immunohistochemistry results of bone marrow samples showed ER (-), PgR (-), HER2 (-), GATA3 (-), and GCDFP (-) (**Figure 2B**). We transfused red blood cells, platelets, and plasma and provided antiviral therapy, symptomatic hemostasis, analgesia, and herbal adjuvant therapy until the definitive diagnosis was achieved.

**Figure 4:**
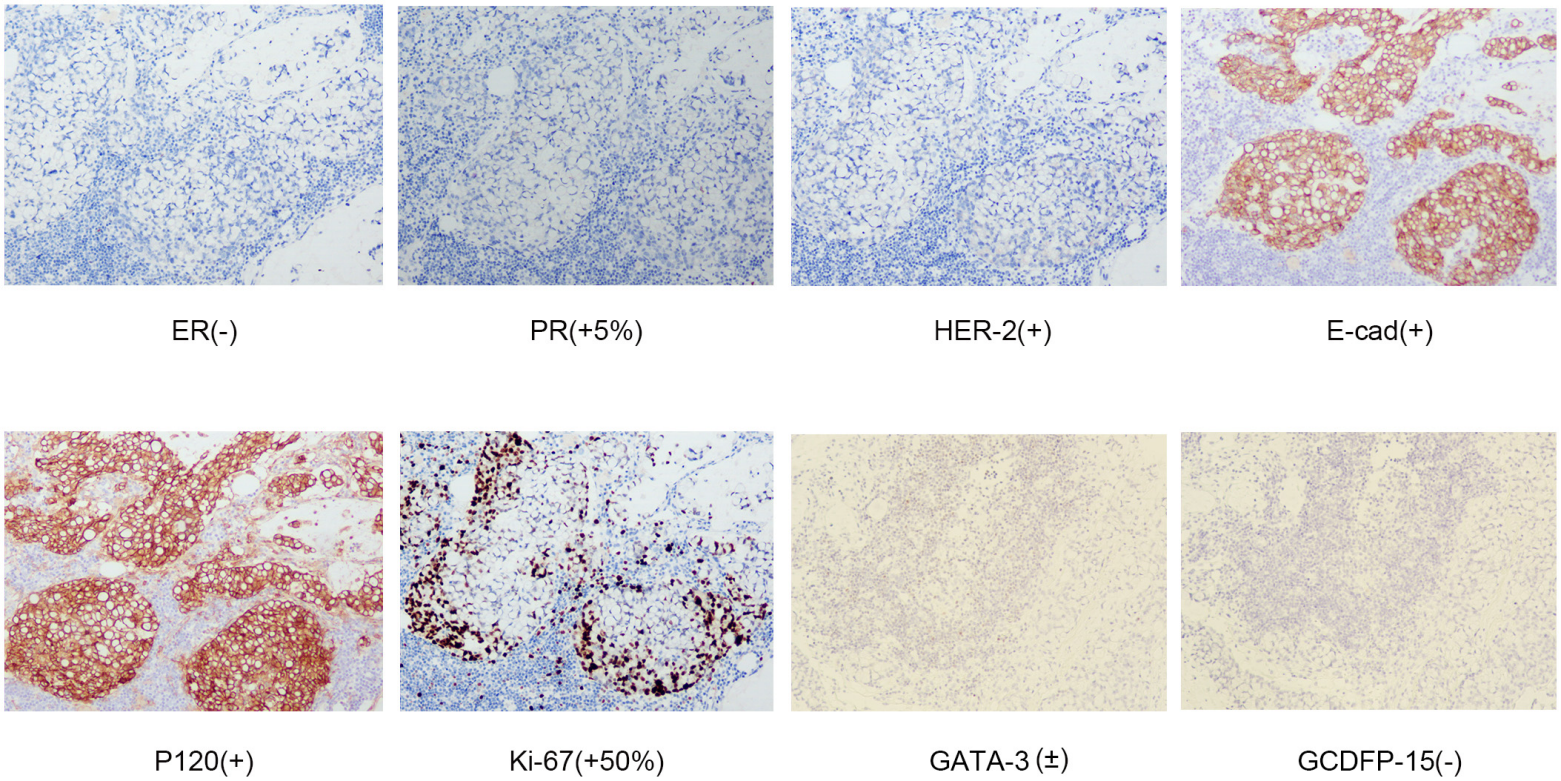
Immunohistochemical staining of a specimen of the right axillary mass in 2024.

Surprisingly, a mass was found near the patient’s right axilla during a physical examination one month later. The mass measured approximately 1.5 cm × 2 cm with no redness, ulceration, or tenderness. A puncture biopsy revealed tumor cells. Subsequently, the patient underwent tumor resection, and the pathology showed invasive adenocarcinoma (**Figures 1G–I**). Immunohistochemistry showed ER (-), PgR (+5%), c-erbB-2 (1+), E-cad (+), GATA-3 (±), GCDFP-15 (-), Ki67 (+ 50%), and p120 (membrane +) (**Figure 4**). Based on all examinations and laboratory results, we diagnosed the patient with invasive ductal carcinoma of the breast with bone marrow metastasis and axillary metastasis. The patient had an Eastern Cooperative Oncology Group (ECOG) physical status score 4. After assessing the patient’s physical condition, we decided on conservative supportive treatment. On April 19, the patient presented with a gradual worsening of pain in the right upper extremity. One week later, the patient once more presented with heavy vaginal bleeding, malaise, fever, and dyspnea following exertion. The treatment was terminated based on the patient’s expressed wishes. She subsequently succumbed to her illness the following day.

## 3 Discussion

DCIS is usually not considered metastatic. However, our patient developed BMM and BMN 11 years after the diagnosis of DCIS, a rare occurrence (16). Clinicopathologic parameters associated with aggressive recurrence and distant metastasis include patient age (<40 years), DCIS size, nuclear grade, presence or absence of consolidated necrosis, histologic type, Ki-67 staining, multifocality, surgical margins, and mode of detection (asymptomatic versus screening test) (17, 18). The patient was 35 at diagnosis, prompted by bloody nipple discharge. No other regular risk factors for distant metastasis were present. Because distant metastases after DCIS are rare and mostly reported as case studies, none of these risk factors were statistically significant. Generally, the mechanisms of recurrence and metastasis in early-stage breast cancer are linked to the completeness of initial treatment, the dormancy and activation of tumor cells, genetic factors, and changes in the tumor microenvironment (19, 20).

Standard treatment for DCIS includes radiotherapy (RT) following breast-conserving surgery (BCS) (21). The NSABP protocol B-17 showed a 12-year local recurrence rate of 32% for DCIS patients treated with resection only and 16% for resection plus radiotherapy (22). Tamoxifen is a selective estrogen receptor modulator (SERM) that is widely used to treat early-stage breast cancer that is hormone receptor-positive (ER+/PgR+) and can significantly reduce the risk of breast cancer recurrence and death (23). Studies have shown that adding tamoxifen to local excision and radiation therapy in ER-positive DCIS patients can reduce the 10-year risk of recurrence (24). However, noncompliance with tamoxifen therapy, especially early discontinuation, may lead to increased recurrence and mortality in breast cancer patients (20, 25). Patients did not take tamoxifen regularly, potentially expanding the recurrence risk.

The tumor microenvironment (TME) plays a crucial role in BMM, promoting tumor cells’ survival, dormancy, and eventual reactivation. Breast cancer cells can reprogram the bone marrow microenvironment, which promotes tumor cell adhesion, angiogenesis, and remodeling of the bone marrow stroma (26). Dormant tumor cells typically survive the successful treatment of the primary tumor and enter a clinically asymptomatic state (27). In the bone marrow, immune cells help form pre-metastatic niches, creating an environment favorable for the survival of DTCs (28, 29). These immune cells secrete pro-inflammatory cytokines that enhance breast cancer tumor cell migration and colonization and mediate the reactivation of dormant DTCs, leading to significant metastasis after long-term dormancy (30). Emerging evidence suggests that COVID-19 can reactivate dormant tumor cells in response to microenvironmental cues, such as inflammatory or immune-mediated signals, leading to tumor progression and metastasis, systemic inflammation, widespread coagulation dysfunction, and multi-organ dysfunction (31). Upon admission, this patient was infected with COVID-19 but was not systematically treated. After this admission, the patient was re-infected with COVID-19, accompanied by fever, elevated D-dimer, and falling platelets. The patient tested positive for the novel coronavirus and had a fever, which conformed to the typical presentation of COVID-19 (32). Subsequently, the patient’s condition deteriorated dramatically. We speculate that the patient’s BMM after 11 years of asymptomatic survival and the rapid development of BMN may be due to immune activation by COVID-19, resulting in a transformation from asymptomatic BMM to symptomatic BMM.

Distant metastasis of breast DCIS in the absence of local lesion recurrence is rare. Previous studies have reported two cases of DCIS where patients developed metastases to the liver, lung, bone, and colon after breast-conserving surgery and endocrine therapy despite the stability of the primary lesion (33, 34). Unlike them, the patient in this case had hardly received any endocrine treatment, and the site of distant metastasis was the bone marrow, which is much more aggressive, has a worse prognosis, and is much rarer. Notably, this patient initially experienced abnormal symptoms, including irregular vaginal bleeding, heavy and prolonged menstrual periods, and later bone pain. As the condition progressed, severe pain (NRS score 8) developed in multiple areas. An increase in vaginal bleeding accompanied each worsening of clinical symptoms. The patient’s fever gradually returned to normal after antiviral treatment, which complicated the diagnosis. Laboratory findings associated with BMN typically reveal anemia, thrombocytopenia, elevated alkaline phosphatase levels, decreased blood calcium, and increased plasma D-dimer. However, these manifestations do not necessarily occur simultaneously (35). Upon admission, the patient presented with anemia and low platelet counts. Subsequent laboratory tests revealed a progressive increase in plasma D-dimer, elevated alkaline phosphatase, decreased blood calcium, and abnormal liver function. Nonetheless, these indicators lack specificity.

Symptomatic bone marrow metastasis (BMM) with a stable primary lesion is rare and prone to misdiagnosis. The patient was admitted to the hospital, and peripheral blood tests showed anemia and a persistent drop in platelets. The initial chest CT examination showed no recurrence or metastasis in the breast, while the PET-CT examination showed diffuse high density in the bone marrow cavity and no metabolic abnormalities in the right breast, armpit, or other areas. These findings made the diagnosis more difficult. In this case, the first step was to rule out hematologic disorders. Bone marrow examination is a high-yield test for identifying BMM from solid tumors (36, 37). In some cases where the primary tumor is occult, immunohistochemical results of bone marrow aspiration and bone marrow biopsy can help identify an unknown primary tumor (36). The first bone marrow biopsy confirmed metastatic cancer of undetermined origin, while further tests ruled out blood disorders. Although BMM is generally straightforward to detect, pinpointing the exact primary site can be challenging. Mammary BMM is commonly associated with bone metastases, and its occurrence without bone metastasis is extremely rare (34). Whole-body CT scans showed no bone metastases, and no cortical bone destruction was observed in the results of the PET-CT. A bone marrow biopsy confirmed metastatic cancer in the bone marrow, yet systemic examination findings remained inconclusive, making the diagnostic process more challenging. The eventual identification of a painless right axillary mass, absent on earlier imaging, was pivotal for diagnosis. By integrating findings from three bone marrow biopsies, PET-CT scans, and the axillary mass, it was concluded that the patient exhibited invasive carcinoma of the breast with BMM and BMN, accompanied by axillary metastasis. Extensive BMN is rare in solid tumors and is highly susceptible to underdiagnosis and misdiagnosis. A retrospective analysis showed that out of 16,651 bone marrow biopsy specimens, only 2 cases of BMN were associated with breast cancer (38). In this case, with an insidious primary disease and nonspecific symptoms, an aggressive multisite bone marrow aspiration biopsy and PET-CT were performed, which provided the prerequisites for the final definitive diagnosis.

Management of bone marrow metastasis remains a clinical challenge due to the lack of established guidelines (39). Due to the rarity of bone marrow metastases from breast cancer, there is a lack of systematic treatment guidelines, and the published literature consists mainly of case reports and studies with small sample sizes. Personalized treatment plans based on the molecular profile of the tumor and the patient’s clinical condition are essential for optimizing outcomes. Chemotherapy is the mainstay of treatment, and weekly paclitaxel therapy is stable and much less toxic. However, bone marrow toxicity of cytotoxic drugs remains an unavoidable problem. Fluorouracil analogs have shown promising efficacy in clinical application and have been reported to be effective for bone marrow metastasis and DIC in gastric cancer (40). Interestingly, Chan, B. reported a case of bone marrow metastasis from triple-negative breast cancer in which the patient developed liver failure after first-line application of albumin-bound paclitaxel, which was then stabilized and controlled by continuous application of 5-FU infusion, followed by oral capecitabine (41). For HER-positive breast cancer bone marrow metastases, adding antibody-drug couplings targeting HER2 prolongs patient survival (42). By searching the literature, we have seen that immunotherapy has shown promising efficacy in bone marrow metastasis of other types of tumors (43). Still, for triple-negative breast cancer, the application has only been reported for metastasis to other sites (44, 45). No studies are related to the application of immune checkpoint inhibitors for BMM. Further studies are expected to address this issue. It has been found that BMM patients’ OS strongly correlates with platelet levels and ECOG scores (34, 46). In this case, the patient was ultimately diagnosed with BMN and had significant suppression of bone marrow hematopoiesis, with symptoms such as grade IV thrombocytopenia (12 × 109/L) and vaginal bleeding. Moreover, the patient was fragile (ECOG 4), and chemotherapy could not be applied in this case. The patient and family agreed to conservative supportive care. Although the patient’s condition improved slightly with plasma transfusion, platelet transfusion, analgesics, anti-infection measures, and herbal medicine, the lack of effective interventions for BMN and the patient’s critical condition led to a deterioration in her condition, and she died the day after being discharged from the hospital. This case highlights the urgent need for further research into effective treatment strategies for BMN associated with breast cancer, especially for patients with severe comorbidities and impaired bone marrow function.

Breast cancer is a remarkably heterogeneous malignant tumor, and its temporal heterogeneity (dynamic changes in molecular characteristics between primary and recurrent foci) and spatial heterogeneity (differences between different metastatic sites) pose significant diagnostic and therapeutic challenges (44, 45). In this case, the patient’s breast cancer progressed from ductal carcinoma *in situ* (ER+/PgR+/HER2+) to invasive ductal carcinoma (ER-/PgR-/HER2-) and showed further molecular phenotypic changes in bone marrow metastasis and axillary metastasis. The first bone marrow biopsy showed metastatic carcinoma and subsequent bone marrow biopsies showed bone marrow necrosis, suggesting a dynamic change in molecular phenotype, culminating in a triple-negative phenotype. The evolution of breast cancer from ductal carcinoma *in situ* to invasive ductal carcinoma is characterized by changes in hormone receptor and HER2 status reflecting the heterogeneity of its molecular phenotype, and mutations in the *BRCA1* gene in the genetic background may play an important role in this process. Elevated expression of the *BRCA1* gene, an essential gene involved in DNA damage repair, cell cycle regulation, and genome stability, is closely associated with an increased risk of early distant metastasis in ER+ breast cancer patients (19, 47). *BRCA1* gene mutations are strongly associated with genomic instability, epithelial mesenchymal transition (EMT), and immune microenvironmental interactions, leading to significant inter- and intratumoral heterogeneity, which affects clinical outcomes and drug resistance (48). Our patient carries a *BRCA1* mutation, and she declined the recommendation for prophylactic mastectomy. The exact time point of the molecular typing change remains uncertain due to the lack of adequate follow-up review in this case, which constitutes a significant limitation.

## 4 Conclusion

We describe a rare case of a female patient who developed symptomatic bone marrow metastasis (BMM) and bone marrow necrosis (BMN) 11 years after the diagnosis of DCIS. DCIS may develop into a tumor that is highly aggressive under certain circumstances. Regular follow-up examinations after systemic therapy are necessary. In breast cancer patients presenting with unexplained anemia, fatigue, fever, bone pain, or abnormal vaginal bleeding while the primary lesion remains stable, the possibility of bone marrow metastasis or bone marrow necrosis should be considered. A bone marrow biopsy should be performed actively, and a PET-CT examination should be performed if necessary to confirm the diagnosis as soon as possible. Early treatment can benefit patient survival.

## Data Availability

The original contributions presented in the study are included in the article/supplementary material. Further inquiries can be directed to the corresponding author.
